# Adaptive servo-ventilation in patients with chronic heart failure and sleep disordered breathing: predictors of usage

**DOI:** 10.1007/s11325-020-02182-2

**Published:** 2020-09-03

**Authors:** Leonie Kolb, Michael Arzt, Stefan Stadler, Katharina Heider, Lars S. Maier, Maximilian Malfertheiner

**Affiliations:** Department of Internal Medicine II, Cardiology and Pneumology, Center for Sleep Medicine, University Medical Center Regensburg, Regensburg, Germany

**Keywords:** Central sleep apnea, Adaptive servo-ventilation, Usage, Adherence, Cheyne Stokes respiration, Sleep stages

## Abstract

**Purpose:**

Adaptive servo-ventilation (ASV) is a therapy designed for patients with central sleep apnea (CSA) and Cheyne Stokes respiration. The aim of this study was to find predictors of ASV usage in patients with CSA in a routine sleep clinic cohort.

**Methods:**

In this retrospective study, consecutive patients in whom ASV therapy was initiated at the University Hospital Regensburg between 2011 and 2015, were analyzed. Analysis included polysomnographies of diagnostic and ASV initiation nights, a phone questionnaire on ASV usage, readout of the ASV device 1 month after initiation (“early ASV usage,” 1 month after ASV initiation), and the readout of the last month before a reappointment date set in 2015 (“late ASV usage,” median 17 months after ASV initiation).

**Results:**

In 69 consecutive patients, the mean early and late ASV usage per night was 4.8 ± 2.5 h and 4.1 ± 3.0 h, respectively. Seventeen months after initiation, 57% of patients used the device ≥ 4 h per night, and of those 91% reported a subjective benefit from ASV therapy. Early ASV usage was significantly associated with late ASV usage (univariable regression: Beta 0.8, 95%CI [0.6; 1.0] *p* < 0.001). In multivariable regression analysis, short duration of slow wave sleep (N3) during diagnostic polysomnography (Beta − 6.2, 95%CI [− 11.0; − 1.5]; *p* = 0.011) and subjective benefit from ASV (Beta 174.0, 95%CI [68.6; 279.5]; *p* = 0.002) were significantly associated with longer late ASV usage.

**Conclusion:**

Early ASV usage predicts late ASV usage. In addition, low slow wave sleep before ASV initiation and subjective benefit from ASV may contribute to higher late ASV usage.

**Electronic supplementary material:**

The online version of this article (10.1007/s11325-020-02182-2) contains supplementary material, which is available to authorized users.

## Introduction

Positive airway pressure therapies such as continuous positive airway pressure (CPAP) and adaptive servo-ventilation (ASV) are important treatments of various forms of sleep disordered breathing (SDB). ASV was designed for patients with central sleep apnea (CSA) and Cheyne Stokes respiration (CSR) [1–3] and is also effective in patients with other types of CSA [4–6]. Since the results of the SERVE-HF trial became public in 2015, showing an increased risk for cardiovascular mortality in the ASV-treated group of patients with chronic heart failure and reduced ejection fraction (HFrEF, EF ≤ 45%), NYHA class II–IV, and predominant CSA [5–8], ASV is contraindicated in this specific patient population [5–7]. However, most of the patients who are treated with ASV in routine clinical care have severe CSA and treatment emergent CSA and a history of heart failure with preserved ejection fraction (HFpEF) [4]. The subgroup in which ASV is contraindicated is small [4], and ASV is still recommended for the treatment of CSA. Recommendations are based on the findings that in patients with CSA, ASV is more efficient in suppressing central apneas and hypopneas [1, 5, 7, 9, 10]. An improvement in quality of life, sleep quality, cardiopulmonary efficiency, and a reduction of elevated natriuretic peptides under ASV therapy were also reported [5, 7, 9, 11–13].

Previous studies support that longer usage of PAP therapy leads to better therapy success [13–15]. For example, longer CPAP usage is associated with improved blood pressure control [15–17] as well as cognitive functions [17, 18]. In most ASV and CPAP studies, sufficient therapy usage is considered to be a usage above 4 h per night [16, 18–23]. The usage of ASV therapy in the previous studies ranged between 3.7 and 5.2 h/night [9–11, 24–26]. Variables predicting usage behavior could be helpful to guide selection of patients for treatment and treatment indication as well as to support patients undergoing ASV initiation in their individual needs and thus optimize therapy usage.

Previous studies evaluating predictors of ASV compliance identified proactive patient management [25] and high early ASV usage as predictors [13] for high late ASV usage. Such studies were either limited by the lack of a full clinical data set [25] or by a specific oligosymptomatic study population of a long-term randomized controlled trial with control arm [13]. Thus, the aim of this study was to find predictors of ASV usage of patients with CSA in a routine sleep clinic cohort.

## Methods

### Patients

**Fig. 1:**
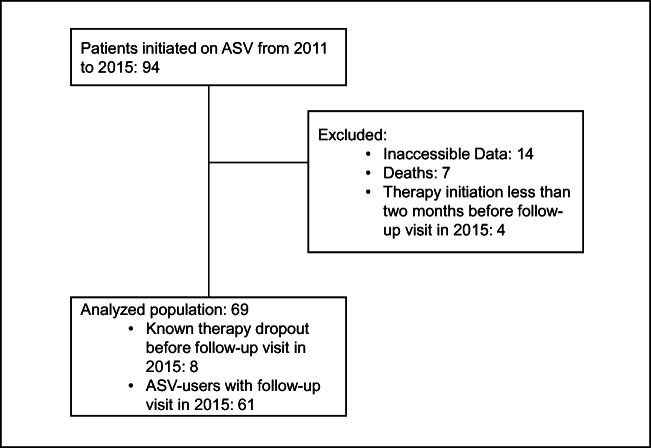
Patient flow chart. *ASV* adaptive servo-ventilation; numerical value: number of patients, follow-up visit: 2015; the total number of patients included in this analysis was 69

This retrospective, monocentric analysis included all consecutive patients, whom ASV therapy has been prescribed between 2011 and May 2015 at the Department of Internal Medicine II at the University Medical Center Regensburg. Exclusion criteria for this analysis were death before follow-up visit in 2015, inaccessible data on late therapy usage at the reappointment date 2015, and initiation of ASV therapy less than 2 months before the reappointment date (Fig. 1).

All patients were contacted between June and October 2015 to reassess the indication for ASV after publication of the results of the SERVE-HF results [8]. Indication for ASV included hypo- or normocapnic CSA in stable heart failure, primary CSA, and treatment emergent CSA. Patients with severe pulmonary disease were excluded. Some patients treated with ASV therapy had heart failure with reduced ejection fraction (≤ 45%), NYHA class II–IV, and predominant CSA, referred to as “risk group” [5, 6, 8]. This analysis was approved by the Ethics Committee of the University of Regensburg (approval no. 15-101-0255) and was conducted in accordance with the principles of Good Clinical Practice and the Declaration of Helsinki.

### Baseline assessment

Assessment included patient records of the first patient visit at the sleep laboratory, including patient characteristics, medication, comorbidities, Epworth sleepiness scale (ESS) score, echocardiography results, diagnostic polysomnography (PSG), and PSG of the ASV initiation night.

### Follow-up assessment

At a follow-up visit in 2015 (median 17 months with an IQR of 16 months after therapy initiation), the usage of the ASV device 1 month after initiation (“early ASV usage”) and of the last month before the reappointment date (“late ASV usage”) were objectively assessed using the readout of the ASV devices. The 8 patients, who did not attend this last follow-up assessment, were known therapy dropouts before follow-up visit in 2015 with a mean late ASV usage of 0.0 h per day. Routine assessment included a questionnaire on subjective ASV usage behavior and subjective benefit from ASV. Subjective benefit was assessed on a dichotomous nominal scale: (1) “yes,” if patients perceived ASV therapy beneficial, and (2) “no,” if patients perceived ASV therapy non beneficial.

### Epworth sleepiness scale

The ESS questionnaire is a validated questionnaire on daytime sleepiness. Scores range from 0 (least sleepy) to 24 (sleepiest). Excessive daytime sleepiness was defined as a score of 11 or higher [27].

### Polysomnography

PSGs of diagnostic and ASV initiation nights were analyzed as prescribed previously [4, 28] according to routine standard criteria. Sleep and associated events were determined according to the American Academy of Sleep Medicine (AASM) Manual 2007 and the following updates [29–32]. The arousal index is the number of interruptions of sleep per hour of sleep. The oxygen desaturation index (ODI) was defined as the number of episodes with oxygen desaturation ≥ 4% per hour of sleep. Patients with a proportion of over 50 % central apneas of total apneas (cAI/AI) were diagnosed with central sleep apnea (CSA). Periodic breathing pattern (Cheyne Stokes respiration—CSR) was diagnosed when both of the following conditions were fulfilled: (1) ≥ 3 consecutive episodes of central apnea and/or hypopnea, separated by a crescendo-decrescendo change in breathing amplitude with a cycle length of at least 40 s (typically lasting 45–90 s) and (2) ≥ 5 central apnea and/or hypopnea episodes per hour associated with the crescendo or decrescendo breathing pattern recorded over a minimum of 2 h of monitoring [30].

### Clinical definitions of central sleep apnea

CSA was defined according to the ERS Task Force statements on central breathing disturbances during sleep [5] and the international classification of sleep disorders [33]. According to the clinical information available, patients were diagnosed with CSA in heart failure if they had HFrEF or HFpEF without documented opioid intake [5]. CSA in stroke was diagnosed with a preceding stroke being the best explanation for the occurrence of CSA. Definitions for treatment emergent CSA included (a) AHI ≥ 5/h and predominantly obstructive respiratory events in the diagnostic PSG; (b) significant resolution of obstructive events and emergence of persistent central events during positive airway pressure treatment with a central AHI of ≥ 5/h and ≥ 50% central events; and (c) the occurrence could not be better explained with another CSA disorder [5]. Drug-induced CSA was diagnosed when CSA occurred in the context of drugs that are known to induce CSA (such as opioids and baclofen). Primary CSA was diagnosed when none of the previously stated causes applied.

### Echocardiography

Echocardiography was routinely performed in all patients prior to PSG to measure left ventricular systolic function and signs of relevant structural heart disease. In the context of the findings of the SERVE-HF trial [3, 8], patients with symptoms and/or signs of heart failure and left ventricular ejection fraction (LVEF) ≤ 45% were classified as HFrEF. Patients with LVEF > 45%, symptoms, and/or signs of heart failure and signs of relevant structural heart disease (left atrial enlargement or diastolic dysfunction) were classified as HFpEF.

### Statistical analysis

Patients were initially stratified into two groups according to late ASV usage: sufficient usage was defined as ASV usage ≥ 4 h, whereas usage < 4 h was defined as insufficient usage. Continuous data are expressed as mean ± standard deviation (SD), unless otherwise stated. To compare usage groups “usage ≥ 4 h” and “usage < 4 h,” continuous variables were compared using a two-sided *t* test, whereas nominal variables were compared by using the chi-square test or, when the expected frequency was < 5, the Fisher’s exact test. For the comparison of diagnostic with ASV initiation night, paired *t* test was used. Delta variables were defined to show the changes of various PSG parameters from diagnostic to ASV initiation night, with the value of the diagnostic night being subtracted from the value of the ASV initiation night. Univariable ANOVA was used to test the differences in the changes from both usage groups “usage ≥ 4 h” and “usage < 4 h.” To assess the effect of the parameters on late usage, uni- and multivariable regression models were applied. A multivariable linear regression model, including all independent variables with *p* < 0.1 in the univariable models, was calculated. In addition, the multivariable model accounted for age, sex, and BMI. A two-sided *p* value < 0.05 was considered statistically significant. Statistical analysis was performed using SPSS Statistic software version 23.0 (IBM, Corp., New York).

## Results

### Patient characteristics

Between 2011 and 2015 in 94 patients, ASV therapy was initiated. Exclusion criteria included death before follow-up visit in 2015, inaccessible data on late therapy usage at the reappointment date 2015 and initiation of ASV therapy less than 2 months before the reappointment date (Fig. 1). From the remaining 69 patients, 61 appeared to the follow-up visit and 8 chose to terminate ASV therapy before the end of 2015 due to discomfort and lack of symptomatic benefit (Fig. 1).

The analysis population consisted predominantly of elderly mildly obese men. The majority fulfilled the diagnostic criteria of heart failure either with reduced or with preserved left ventricular ejection fraction (Table 1). The vast majority of patients had echocardiographic abnormalities such as left atrial enlargement, left ventricular hypertrophy, and/or diastolic dysfunction (Table 1).

**Table 1:** Patient characteristics at baseline

	Analysis population
*n* (%)	**69 (100 %)**
Age [years]	69 ± 10
Body mass index [kg/m^2^]	31 ± 6
Male sex [n (%)]	66 (96%)
Diagnosis of heart failure	
No heart failure [*n* (%)]	14 (20%)
HFrEF [*n* (%])	17 (25%)
HFpEF [*n* (%)]	38 (55%)
NYHA-classification (in HFrEF and HFpEF patients)	
NYHA I, II [*n* (%)]	34 (62%)
NYHA III, IV [*n* (%)]	21 (38%)
Ejection fraction [%]	49 ± 14
Left atrial enlargement [*n* (%)]	39 (57%)
Left ventricular hypertrophy [*n* (%)]	43 (62%)
Diastolic dysfunction [*n* (%)]	19 (28%)

Data are presented as mean ± standard deviation or *n* (%). *NYHA* New York Heart Association functional class, *HFrEF* heart failure with reduced ejection fraction, *HFpEF* heart failure with preserved ejection fraction

The analysis population had severe predominantly CSA with a mildly reduced mean SaO2. Sleep was fragmented as well as sleep efficiency (SE); N3 and REM sleep were reduced. Sleep onset latency was prolonged (Table 2). Patients reported a moderate degree of subjective daytime sleepiness (Table 2).

**Table 2:** Diagnostic polysomnography

	Analysis population
*n* (%)	**69 (100%)**
Sleep onset latency, *min*	25.0 [14.3; 41.1]*
Sleep efficiency, *%*	73 ± 16
Apnea-hypopnea index, *n/h*	48 ± 20
Apnea index, *n/h*	34 ± 22
Central apnea index, *n/h*	20 ± 17
Min. SaO_2_, *%*	77 ± 10
Mean SaO_2_, *%*	92 ± 2
Oxygen desaturation index, *n/h*	44 ± 18
Respiratory arousal index, *n/h*	38 ± 16
Sleep stage N1, *%*	31 ± 17
Sleep stage N2, *%*	45 ± 14
Low wave sleep (N3), *%*	12 ± 9
Rapid-eye-movement sleep, *%*	12 ± 6
ESS baseline	9 ± 5

Data are presented as mean ± standard deviation or *n* (%). *Data are presented as median [interquartile range]

### Adaptive servo-ventilation usage

**Fig. 2:**
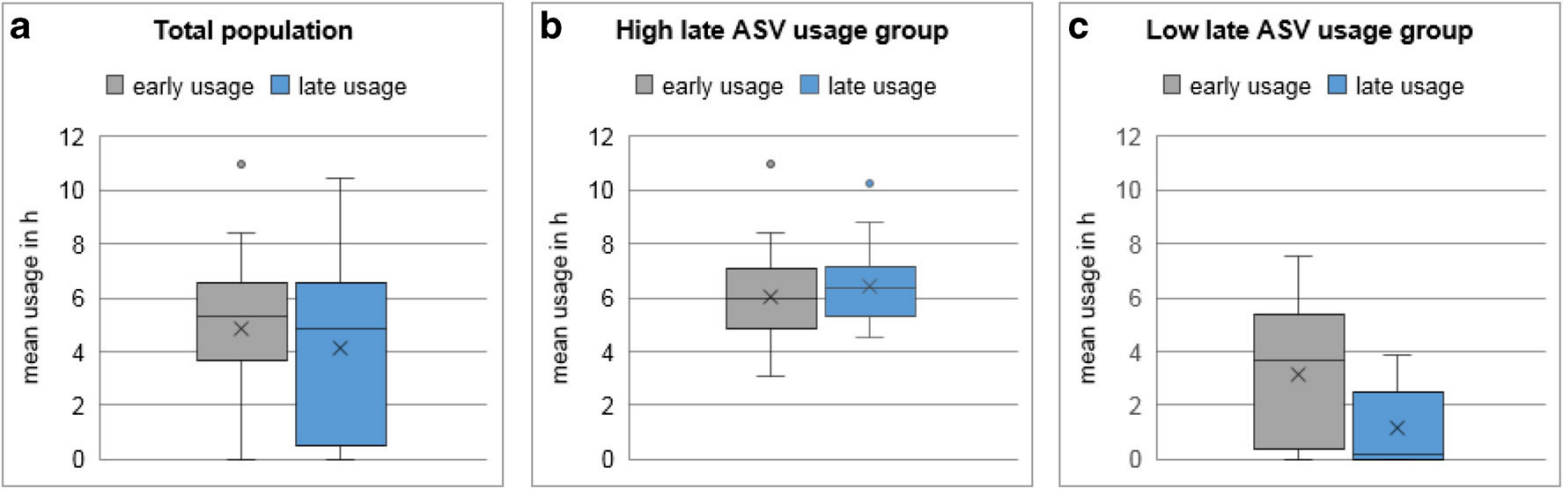
**a** Box plot shows in the total population a significant fall from early to late ASV usage (4.8 ± 2.5 h versus 4.1 ± 3.0 h, *p* < 0.001). The total range of early and late ASV usage was 0 to 11 h and 0 to 10 h per night, respectively. **b** Patients of the high late ASV usage group had similar early and late usage (6.1 ± 1.7 vs. 6.4 ± 1.4, *p* = 0.399). **c** Patients of the low late ASV usage group had a further fall from early to late ASV usage (3.2 ± 2.6 vs. 1.2 ± 1.4 h, *p* < 0.001). Box plot shows median usage (horizontal line) with IQR. Whiskers show maximum/minimum value still within 1.5 IQR of upper/lower quartile. Outliners are depicted as dots. Mean usage is depicted as cross. *Early usage:* ASV usage in the first month after therapy initiation, *late usage:* ASV usage in the last month before follow-up visit in 2015

The mean early ASV usage was 4.8 ± 2.5 h, and the mean late usage was 4.1 ± 3.0 h in the total population (Fig. 2a). Eleven patients (15.9%) stopped ASV therapy within the follow-up period (median 17 months). Patients were stratified into a group with late ASV usage ≥ 4 h (high late ASV usage; *n* = 39, 57%) and a group with late ASV usage < 4 h (low late ASV usage: *n* = 30, 44%, Table 1. Patients with high late ASV usage had similar early and late usage (6.1 ± 1.7 vs. 6.4 ± 1.4, *p* = 0.399; Fig. 2b), while patients with low late ASV usage had a further fall from early to late ASV usage (3.2 ± 2.6 vs. 1.2 ± 1.4 h, *p* < 0.001; Fig. 2c). Among patients with documented high early ASV usage (≥ 4 h), the proportion of high late ASV usage was significantly greater compared with low late ASV usage (72 vs. 28%).

### Patient characteristics according to late adaptive servo-ventilation usage

Thirty-nine patients used their device ≥ 4 h (57%, high late ASV usage), and 30 patients used their device < 4 h (44%, low late ASV usage). Groups did not differ with respect to demographic parameters (eTable 1). In the low late ASV usage group, the proportion of patients with AF and ischemic cardiomyopathy was significantly higher compared with the high late ASV usage group (eTable 1).

### Sleep parameters and applied pressures

**Fig. 3:**
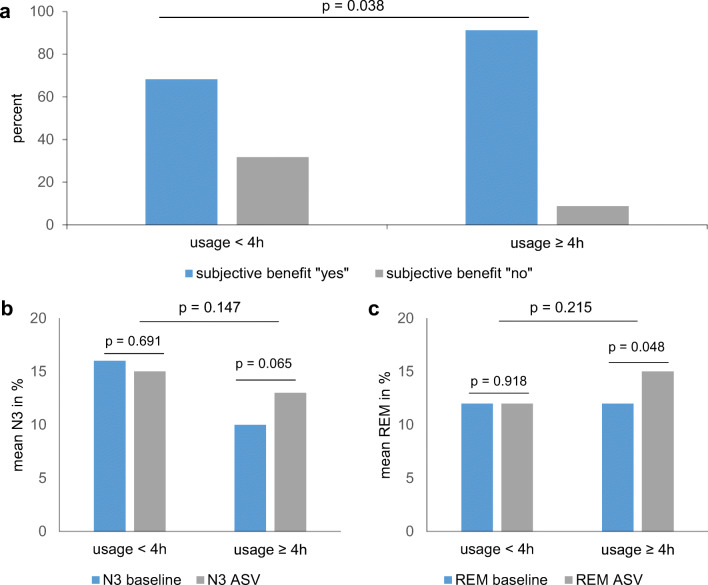
Comparison of the usage groups. *Panel a* compares the subjective benefit in the ASV usage groups. Subjective benefit was assessed at the reappointment date with dichotomous nominal scale: “yes” if patients perceived ASV therapy beneficial, “no” if patients perceived no benefit from ASV therapy. Patients with usage ≥ 4 h reported significantly more often subjective benefit. *Panel b and c* compare the ASV usage groups at diagnostic and ASV initiation night. *Panel b* shows the tendency of prolongation of slow wave sleep in patients with ASV usage ≥ 4 h. *Panel c* shows the significant prolongation of REM sleep in usage group “usage ≥ 4 h”. *N3* slow wave sleep, *REM* rapid-eye-movement sleep

Comparison of the usage groups with respect to the PSGs of diagnostic and ASV initiation night showed no significant differences (Fig. 3). In the group “usage < 4 h,” sleep efficiency showed a reduction in the ASV night (Fig. 3). Groups had similar pressure settings 1 month after therapy initiation (mean EPAP: usage < 4 h: 8.2 ± 2.6, usage ≥ 4 h: 7.6 ± 2.1 *p* = 0.890; mean maximum inspiratory pressure support usage < 4 h: 6.5 ± 4.3, usage ≥ 4 h: 7.1 ± 4.5 *p* = 0.624).

### Subjective benefit

Patients who stated a subjective benefit from ASV therapy used their device significantly more (*p* value = 0.038) (Fig. 3) with a total of 82 % of the patients reporting subjective benefit from the therapy. In mean patients showed no excessive signs of daytime sleepiness prior to therapy with the mean ESS baseline being 9 ± 5 points (Table 2). Daytime sleepiness showed no influence on ASV usage behavior (eTable 1).

### Regression models

A univariable regression model was used for variables showing an association with late ASV usage: early usage (*p* ≤ 0.001), the absence of AF (*p* = 0.021), the absence of ischemic cardiomyopathy (*p* = 0.043), and subjective benefit (*p* ≤ 0.001) proofed to be significant predictors for late usage. An improvement of sleep quality was associated with late usage (Delta REM: *p* = 0.036; Table 3). Left ventricular ejection fraction was not associated with late ASV usage (Beta coefficient [95% confidence interval]: − 0.07 [− 5.134, 3.49], *p* = 0.701).

**Table 3:** Predictors for long-term usage of adaptive servo-ventilation—univariable regression model*

Variable	Beta (95%CI) [min]	*p* value
Age (baseline) [years]	− 2.5 (− 6.9; 1.9)	0.264
Body mass index [kg/m^2^]	− 2.193 (− 10.077; 5.690)	0.580
Sex [male vs female]	145.591 (− 63.549; 354.731)	0.169
Atrial fibrillation [yes vs no]	− 98.295 (− 181.515; − 15.076)	**0.021**
Ischemic cardiomyopathy [yes vs no]	− 87.351 (− 171.978; − 2.725)	**0.043**
NYHA [I, II vs III, IV]	14.314 (− 75.619; 104.246)	0.752
HFrEF vs. not	16.791 (− 69.218; 102.799)	0.697
Type of CSA [CSA in HF vs primary CSA vs treatment emergent CSA]	60.228 (− 16.972; 137.427)	0.123
Epworth sleepiness scale score	− 0.550 (− 11.001; 9.901)	0.916
Sleep efficiency	− 1.958 (− 5.079; 1.163)	0.213
Apnea-hypopnea index	0.041 (− 2.349; 2.431)	0.973
Central apnea index	− 0.025 (− 2.914; 2.864)	0.986
Slow wave sleep (N3) [%]	− 4.466 (− 9.426; 0.493)	0.076
Rapid-eye-movement sleep [%]	− 3.432 (− 11.454; 4.591)	0.394
Delta Epworth sleepiness scale score	− 0.169 (− 15.332; 14993)	0.982
Delta sleep efficiency	1.580 (− 0.997; 4.158)	0.223
Delta apnea-hypopnea index	0.151 (−2.518; 2.820)	0.910
Delta central apnea index	0.622 (− 2.271; 3.515)	0.667
Delta slow wave sleep	2.187 (− 2.262; 6.636)	0.328
Delta rapid-eye-movement sleep	5.504 (0.377; 10.632)	**0.036**
Subjective benefit [yes vs no]	191.300 (82.182–300.418)	**0.001**
Mean EPAP [cmH_2_O]	− 0.992 (− 21.782–19.798)	0.924
Mean IPAP [cmH_2_O]	2.379 (− 16.150− 20.908)	0.797

Values with *p* ≤ 0.01 are printed in bold. *Beta* beta-coefficient, *CI* confidence interval, *NYHA* New York Heart Association functional class, *HFrEF* heart failure with reduced ejection fraction, *HFpEF* heart failure with preserved ejection fraction, *CSA* central sleep apnea, *EPAP* expiratory positive airway pressure, *PS* pressure support, *IPAP* inspiratory positive airway pressure. All Delta values are value from ASV initiation night minus value from diagnostic night—except Delta ESS: value from follow-up visit minus value from diagnostic night. *Long-term usage = mean nocturnal use of adaptive servo-ventilation in minutes

In multivariable analysis, short duration of slow wave sleep at baseline and subjective benefit predicted longer late ASV usage, when the model was adjusted for all potential predictors with *p* < 0.1 in the univariable analysis (Table 3), as well as age, BMI, and sex (Table 4). Early usage could not be included into that model because of multi-correlation with the subjective benefit. None of the potential predictors for ASV usage such as age, sex, BMI, subjective benefit, atrial fibrillation, ischemic cardiomyopathy, N3 in diagnostic PSG, change of rapid-eye-movement sleep, and subjective benefit from ASV was significantly associated with early ASV usage ≥ 4 h (multivariable binary regression analysis, *p* > 0.05 for each potential predictor). Only N3 in diagnostic PSG was significantly associated with late ASV usage ≥ 4 h (multivariable binary regression analysis, Beta [95% CI]: 0.891 [0.798; 0.995]; *p* = 0.04).

**Table 4:** Predictors for long-term usage to adaptive servo ventilation—multivariable regression model*

Variables	Beta (95%CI)	*p* value
Age [years]	− 2.405 (− 7.459; 2.650)	0.339
Body Mass Index [kg/m^2^]	− 1.639 (− 10.903; 7.625)	0.720
Male sex	64.335 (− 138.140; 266.811)	0.521
Atrial fibrillation [yes vs no]	− 82.479 (− 178.341; 13.384)	0.089
Ischemic cardiomyopathy [yes vs no]	− 73.782 (− 163.371; 15.807)	0.103
N3 in diagnostic PSG [%]	−5.874 (− 10.587; − 1.161)	**0.016**
Delta rapid-eye-movement sleep	1.703 (− 3.377; 6.783)	0.499
Subjective benefit from ASV [yes vs no]	147.800 (39.988; 255.612)	**0.009**
Model summary	R^2^ 0.546; F = 4.50	

Values with *p* ≤ 0.05 are printed in bold. *Beta* beta-coefficient, *CI* confidence interval, *N3* slow wave sleep. Delta is value from ASV initiation night minus value from diagnostic night. *Long-term usage = mean nocturnal use of adaptive servo-ventilation in minutes

In order to assess the correlation between AHI at baseline/arousal index at baseline and slow wave sleep/non-REM sleep, respectively, simple linear regression models were performed. High AHI and high arousal index at baseline correlated significantly with low percentage of slow wave sleep at baseline (*R*^2^ 0.119, *p* = 0.016 and *R*^2^ 0.262, *p* < 0.001), whereas there was no significant correlation between AHI and arousal index at baseline and percentage of REM sleep at baseline (*R*^2^ 0.013, *p* = 0.436 and *R*^2^ 0.020, *p* = 336). High central apnea index was neither significantly associated with low percentage of slow wave sleep nor with REM sleep at baseline (*R*^2^ 0.003 and *R*^2^ 0.028; *p* > 0.05 for both linear regression models).

## Discussion

This analysis confirmed that high early usage of ASV predicts late ASV usage. In addition, this analysis detected possible predictors of late ASV usage in a sample of patients with chronic heart failure: short duration of slow wave sleep at diagnostic PSG and the subjective benefit from ASV therapy.

In accordance with previous CPAP [34–36] and ASV [37] studies, high early usage was a strong predictor of high late usage (*p* < 0.01). The consistency of this finding in various analyses underlines how early on (early usage was defined from 3 days to 1 month in different analyses) a usage pattern is adopted and underlines the importance of a diligent adaption to the novel therapy as outlined in guidelines for the management of SDB [6]. Attention to comfort with the face mask and the applied positive airway pressure as well as proper communication between the patient and the health worker from the beginning of the therapy on could be a key in preventing patients from dropping out of therapy. Also new strategies as the telemedicine-based proactive patient management, which initiates patient contact, information sharing, and education through a cloud-based remote monitoring system, could be of use to improve therapy usage [38, 39]. In this analysis as well as in the ASV study of Perger et al. [37] in patients with heart failure and sleep apnea and a recent study by van Ryswyk et al. [36] in patients with obstructive sleep apnea and cardiovascular disease with CPAP therapy, adherence at 1 month was the strongest predictor of long time adherence.

Two to 6 weeks after PAP initiation could be a crucial time period during which usage should be evaluated, and patients should be monitored more closely to identify patients with a higher risk of therapy drop out and/or low therapy usage [6, 36, 37]. The fall of daily usage in the present analysis (4.8 to 4.1 h) was similar to the findings from the ASV group of the SERVE-HF trial in patients with HFrEF and CSA with a mean usage of 4.1 h at 2 weeks and 3.4 h at 24 months [24].

The second very strong predictor of late usage was subjective benefit. The concordance of subjective benefit and therapy usage is easily understood. It is plausible that patients who experience a therapy as beneficial are more likely to continue with it. To assess a subjective benefit could therefore be helpful in predicting ASV usage. In this analysis, 82% of the collective felt a beneficial effect of ASV therapy. This could be interpreted as a good sign for ASV therapy in patients in HF (keeping in mind that the subgroup of HF patients in whom ASV is contradicted is fairly small [4]) although a possible placebo effect cannot be ruled out.

Seventy-nine percent of the patients with CSA who are treated with ASV suffer from impaired cardiac function [4]. Patients with heart failure and either OSA [40] or CSA [9, 41] are usually not sleepy [42]. Compared with the general population with a similar degree of OSA heart failure, patients have a longer sleep onset latency, shorter total sleep time, and a lower ESS [40]. One mechanism might be that heart failure patients with sleep-disordered breathing show increased sympathetic nerve activity in comparison with those without [43]. It is a consistent finding in heart failure populations that sleep quality and changes in sleep quality do not strongly correlate with daytime sleepiness [12, 44, 45]. The finding that daytime sleepiness shows no influence on ASV-usage is in accordance with a small study from Philippe et al. [9].

The influence of a subjective benefit on therapy usage is underlined by the finding of low slow wave sleep at diagnostic polysomnography predicting high late usage. In central sleep apnea, low N3 percentage is a sign that CSA is disrupting sleep [13]. This is underlined by the significant correlation of high AHI and high arousal index with low slow wave sleep at baseline in this sample (*R*^2^ 0.119, *p* = 0.016 and *R*^2^ 0.262, *p* < 0.001). Central sleep apnea mainly occurs in non-REM sleep [46] (underlined by the fact that there was no significant correlation between AHI/arousal index with percentage of REM sleep (*R*^2^ 0.013, *p* = 0.436 and *R*^2^ 0.020, *p* = 336). With slow wave sleep being the most important non-REM sleep phase for nocturnal recreation [13, 47], low slow wave sleep at baseline being a predictor for late usage might be interpreted as a sign that patients who find greater relief with their therapy are more likely to continue with it [35]. This interpretation goes hand in hand with earlier findings in CPAP compliance studies in which sleep efficiency at titration night [48] and lower percentage of N2 sleep with higher percentage of REM sleep at initiation night [35] was found in good therapy users. In this analysis, tendencies in the usage group ≥ 4 h showed more impaired sleep structure at baseline, which improved on ASV. In contrast, in the usage group < 4 h, a worsening of sleep efficiency from diagnostic to initiation night was observed. In accordance with these results, Lewis et al. showed that patients who reported problems at their first night of CPAP later used their therapy less [49]. Collen et al. and Lettieri et al. report on an association between use of sedative hypnotics in the titration night and longer TST and higher sleep efficiency, which was also a significant predictor of higher short term CPAP adherence [50, 51].

### Clinical perspective

Variables predicting usage behavior could be helpful in guiding the selection of patients for ASV treatment and for extended proactive patient management in order to optimize device usage [39], bearing in mind that longer usage of PAP treatment leads to greater therapy success [13–15]. The present data underscore that early usage of ASV predicts long-term usage. Thus, early feedback and patient support, possibly with the use of telemedicine based proactive patient management [38, 39], are warranted. Patients with impaired sleep structure (shorter slow wave sleep), with the potential to improve sleep, and those with subjective benefit from ASV therapy have higher long-term usage of ASV: In those with normal sleep structure before therapy and those patients without subjective benefit from ASV treatment, indication should be thoroughly reevaluated.

### Limitations

This retrospective analysis is subject to some limitations. Not all possible predictors of ASV usage were investigated—especially psychological factors [52] have not been analyzed. Also, some physiological parameters such as lung function and blood gas measures were not systematically assessed. Some known predictors for positive airway pressure intolerance such as high nasal resistance [17, 18], uncomfortable mask [19–21], financial aspects, loss of intimacy with the bed partner [22], and claustrophobia [22, 23] were not systematically assessed. As this is an observational study, causal effects cannot be proven, and selection bias can occur. Because of the patients forming part of everyday clinical practice, equal conditions could not be provided: different devices (ResMed or Respironics from Phillips), different types of masks [53] might have influenced usage behavior. On the other hand, the present study presents a real-life clinical setting. A further limitation is that the timespan between diagnostic PSG, ASV initiation, and reappointment date differed in the studied population. But, since the median time from ASV initiation to the reappointment date was 17 months, we considered it long enough to be able to compare late usage. In addition, it is possible that our sample of 69 patients is too small to detect all modulators of late ASV usage. The night-to-night variability of severity of SDB and sleep quality may have diluted the findings in the present analysis, since in clinical routine, rarely repeated polysomnographies are performed.

## Conclusion

Early ASV usage is a strong predictor of late ASV usage. In addition, low slow wave sleep during diagnostic polysomnography and subjective benefit may contribute to high late ASV usage. Findings should be confirmed in analyses of major ongoing prospective registries of ASV such as READ-ASV (Phase I and II, NCT03032029, NCT04331821) or autoSVREGDE (NCT03421704).

## Electronic supplementary material

ESM 1 (DOCX 20 kb)

## Data Availability

Data are available upon request.
