# Supplementary material for: Adaptive servo-ventilation in patients with chronic heart failure and sleep disordered breathing: predictors of usage
Source: Sleep Breath. 2020 Sep 3;25(2):1135–45. doi: 10.1007/s11325-020-02182-2 (PMC8195885; doi:10.1007/s11325-020-02182-2)
Supplement: Supplementary file 1 — (DOCX 20 kb) [file 11325_2020_2182_MOESM1_ESM.docx]

**Adaptive servo-ventilation in patients with chronic heart failure and sleep disordered breathing: predictors of usage**

**Journal: Sleep and breathing**

Leonie Kolb^1*^; Michael Arzt^1^; Stefan Stadler^1^; Katharina Heider^1^; Lars S. Maier^1^; Maximilian Malfertheiner^1^

^1^ Department of Internal Medicine II, Cardiology and Pneumology, Center for Sleep Medicine, University Medical Center Regensburg, Regensburg, Germany

Corresponding author:

Prof. Dr. med. Michael Arzt

Email: michael.arzt@ukr.de

Phone: +49 941 9447281

Facsimile: +49 941 9447282

Mobile: +49 1708088793

**eTable 1:** **Patient characteristics divided by late ASV usage duration**

|  | Low late ASV usage  (< 4h/day) | High late ASV usage  (≥ 4h/day) | p-value |
| --- | --- | --- | --- |
| n (%) | **30 (43%)** | **39 (57%)** |  |
| Age [years] | 70 ± 9 | 68 ± 10 | 0.371 |
| Body Mass Index [kg/m^2^] | 31 ± 5,8 | 30,8 ± 5,6 | 0.854 |
| Male sex [n (%)] | 30 (100%) | 36 (92%) | 0.252 |
| Riskgroup [n (%)] | 4 (15%) | 4 (13%) | 1.000 |
| ESS baseline | 9 ± 5 | 8 ± 4 | 0.665 |
| Heart failure (HF) |  |  |  |
| NYHA I,II [n (%)] | 20 (67%) | 24 (62%) | 0.660 |
| NYHA III,IV [n (%)] | 10 (33%) | 15 (39%) |  |
| Ejection fraction [%] | 49 ± 11 | 49 ± 16 | 0.952 |
| HFrEF [n (%]) | 7 (27 %) | 10 (30%) | 0.355 |
| HFpEF [n (%)] | 19 (73 %) | 20 (61%) |  |
| No HF [n (%)] | 0 (0%) | 3 (9%) |  |
| Left Atrial Enlargement [n (%)] | 19 (73%) | 20 (69%) | 0.737 |
| Left Ventricular Hypertrophy, [n (%)] | 22 (85%) | 21 (75%) | 0.381 |
| Diastolic dysfunction, [n (%)] | 7 (27%) | 12 (43%) | 0.221 |
| Diagnosis CSA |  |  |  |
| CSA in HF [n (%)] | 12 (63%) | 12 (48%) | 0.360 |
| Primary CSA [n (%)] | 0 (0%) | 3 (12%) |  |
| Treatment emergent CSA [n (%)] | 7 (37%) | 10 (40%) |  |
| History |  |  |  |
| Atrial fibrillation [n (%)] | 19 (63%) | 14 (36%) | **0.024** |
| Cheyne Stokes [n (%)] | 16 (59%) | 16 (52%) | 0.559 |
| Ischemic cardiomyopathy [n (%)] | 19 (63 %) | 11 (28%) | **0.004** |
| Diabetes mellitus [n (%)] | 15 (50%) | 13 (33%) | 0.162 |
| Hyperlipidaemia [n (%)] | 24 (80%) | 26 (67%) | 0.219 |
| Arterial hypertension [n (%)] | 26 (87%) | 28 (72%) | 0.138 |
| CHF therapy |  |  |  |
| ICD or pacemaker [n (%)] | 6 (20%) | 9 (23%) | 0.759 |
| ACE-inhibitor/  AT1-antagonist [n (%)] | 18 (60%) | 17 (44%) | 0.176 |
| ß-blocker [n (%)] | 22 (73%) | 24 (62%) | 0.303 |
| Lipid-lowering agent [n (%)] | 20 (67%) | 23 (59%) | 0.513 |

Data are presented as mean ± standard deviation or n (%). P-values compare the two groups by t-tests, chi-square or fisher-exact tests respectively and were considered significant when p ≤ 0.05. Significant values are presented in bold. *Abbreviations:* *NYHA* New York Heart Association functional class, *HFrEF* Heart Failure with reduced Ejection Fraction, *HFpEF* Heart Failure with preserved Ejection Fraction.

**eTable 2: Pressure Settings at ASV initiation**

|  | Total Sample | SE improved | SE worsened | p-value |
| --- | --- | --- | --- | --- |
| Delta SE | **-4.1 ± 18.0** | **12.4 ± 10.4** | **-15.7 ± 12.2** |  |
| EPAP min | 5.9 ± 1.9 | 5.9 ± 1.9 | 5.7 ± 1.2 | 0.560 |
| EPAP max | 12.3 ± 2.7 | 12.3 ± 2.7 | 10.9 ± 1.9 | **0.047** |
| PS min | 0.2 ± 0.7 | 0.15 ± 0.7 | 0.5 ± 1.3 | 0.217 |
| PS max | 9.9 ± 2.6 | 9.9 ± 2.6 | 10.5 ± 2.5 | 0.444 |

Data are presented as mean ± standard deviation or n (%). *SE*: Sleep efficiency, improved/ worsened: patients with positive/ negative Delta SE, *EPAP* Expiratory Positive Airway Pressure, *PS* pressure support. All Delta values are value from ASV initiation night minus value from diagnostic night.
